# Crucial role of autophagy in propofol-treated neurological diseases: a comprehensive review

**DOI:** 10.3389/fncel.2023.1274727

**Published:** 2023-10-25

**Authors:** Sicong Yu, Jian Liao, Xuezheng Lin, Yu Luo, Guangtao Lu

**Affiliations:** ^1^Department of Anesthesiology, Taizhou Central Hospital (Taizhou University Hospital), Taizhou, China; ^2^Department of Nephrology, Jiaxing Hospital of Traditional Chinese Medicine, Jiaxing, China

**Keywords:** propofol, neurological disorders, autophagy, treatment, mechanism

## Abstract

Neurological disorders are the leading cause of disability and death globally. Currently, there is a significant concern about the therapeutic strategies that can offer reliable and cost-effective treatment for neurological diseases. Propofol is a widely used general intravenous anesthetic in the clinic. Emerging studies demonstrate that propofol exerts neuroprotective effects on neurological diseases and disorders, while its underlying pathogenic mechanism is not well understood. Autophagy, an important process of cell turnover in eukaryotes, has been suggested to involve in the neuroprotective properties developed by propofol. In this narrative review, we summarized the current evidence on the roles of autophagy in propofol-associated neurological diseases. This study highlighted the effect of propofol on the nervous system and the crucial roles of autophagy. According to the 21 included studies, we found that propofol was a double-edged sword for neurological disorders. Several eligible studies reported that propofol caused neuronal cell damage by regulating autophagy, leading to cognitive dysfunction and other neurological diseases, especially high concentration and dose of propofol. However, some of them have shown that in the model of existing nervous system diseases (e.g., cerebral ischemia-reperfusion injury, electroconvulsive therapy injury, cobalt chloride-induced injury, TNF-α-induced injury, and sleep deprivation-induced injury), propofol might play a neuroprotective role by regulating autophagy, thus improving the degree of nerve damage. Autophagy plays a pivotal role in the neurological system by regulating oxidative stress, inflammatory response, calcium release, and other mechanisms, which may be associated with the interaction of a variety of related proteins and signal cascades. With extensive in-depth research in the future, the autophagic mechanism mediated by propofol will be fully understood, which may facilitate the feasibility of propofol in the prevention and treatment of neurological disorders.

## Introduction

The nervous system plays an important role in regulating and controlling the physiological functions of the human body, enabling the body to adapt to the changing external environment. It is composed of neurons and glia. Neurological diseases will occur when the structure and function of neurons are impaired or the brain tissue is partially hypoxic, such as cerebral infarction, epilepsy, Alzheimer’s disease (AD), Parkinson’s disease (PD), and perioperative neurocognitive disorder (PND), leading to the dysfunction of language, movement, emotion, and memory (Salter and Stevens, 2017). With the aging of society, neurological diseases seriously endanger human health with a high incidence rate and mortality. More than 750 million people suffering from neurological disorders around the world, which accounts for the majority of disabilities and deaths. But we still know little about the pathogenesis and treatment of neurological diseases. Consistently, great efforts have been made to research the pathogenesis and mechanism to prevent and treat Neurological diseases. In the current research, several hypotheses have been widely concerned, such as apoptosis, autophagy, oxidative stress, and neuroinflammation (Gao and Hong, 2008; W. W. Chen et al., 2016; Singh et al., 2019; Uzdensky, 2019; Park et al., 2020).

Propofol, an alkyl phenol derivative, with the molecular formula C₁₂H₁₈O, is a widely used general intravenous anesthetic in the clinic because of its rapid onset and recovery, such as painless treatment, surgical anesthesia, sedation and maintenance of critically ill patients (including adults and children) in intensive care unit (ICU) (Wehrmann and Triantafyllou, 2010; X. H. Chen et al., 2016; Soliman et al., 2017). It exerts sedative and hypnotic effects via activation of central amino acid receptors γ-aminobutyric acid (GABA) just like other Intravenous general anesthesia drugs (Collins, 1988; Hara et al., 1994). Propofol can mitigate acute renal injury caused by myocardial ischemia-reperfusion injury and sepsis due to its antioxidant capacity (Jin et al., 2009; Wang et al., 2009; Hsing et al., 2011). Nevertheless, we are still not sure about the specific effect of propofol on the nervous system. Studies have shown that propofol can improve brain tissue damage, reduce infarct area, and have neuroprotective effects in patients with cerebral hemorrhage and acute cerebral ischemia (Gelb et al., 2002; Cui et al., 2013; Shi et al., 2015; Zhang et al., 2016). It has also been used to treat patients with chronic insomnia to improve their sleep quality of sleep and life (Xu et al., 2011). However, several studies found that long-term or high dosages of propofol can reduce the activity of neurons and cause developmental neurotoxicity (Bosnjak et al., 2016; Wang et al., 2016; Liu et al., 2019). Ever-increasing evidence reveals that propofol affects the nervous system by regulating autophagy, but the mechanism is still unclear (Cui et al., 2012; Qiao et al., 2017).

Autophagy is an important process of cell turnover in eukaryotes. In this process, some damaged proteins or organelles are wrapped by autophagic vesicles with double membrane structure and then sent to lysosomes (animals) or vacuoles (yeast and plants) for degradation and recycling (Mizushima et al., 2002). Imbalanced autophagy can cause a series of neurological diseases by damaging hippocampal neurons and affecting the formation of spatial memory (Menzies et al., 2017; Hylin et al., 2018). But under physiological conditions, autophagy can promote cell survival via the regulation of energy metabolism and maintenance of cellular homeostasis (Li et al., 2014). Since the extensive clinical application of propofol, it is important to clarify its protective and toxic mechanisms to the nervous system. Autophagy may be one of the regulatory factors in the action of propofol affecting the nervous system. To the author’s knowledge, no comprehensive review has been discussed on the relationship between the propofol-mediated autophagy pathway and the affection of the nervous system. Therefore, it is necessary for us to discuss this issue in time. In this review, we summarized the molecular and biological functions of autophagy in propofol-mediated neuroprotection.

### Literature search

A systematic search was applied to detect the eligible studies from four common databases, including MEDLINE, OVID, Cochrane Library, and Web of Science to screen related studies prior to November 1, 2022. Only studies reported in English were considered to be eligible. The following search strategy was employed in PubMed: [(((((((((((((((((((((“Propofol” [Mesh]) OR (2,6-Diisopropylphenol)) OR (2,6 Diisopropylphenol)) OR (2,6-Bis(1-methylethyl)phenol)) OR (Disoprofol)) OR (Diprivan)) OR (Disoprivan)) OR (Fresofol)) OR (ICI-35,868)) OR (ICI 35,868)) OR (ICI35,868)) OR (ICI-35868)) OR (ICI 35868)) OR (ICI35868)) OR (Ivofol)) OR (Propofol Fresenius)) OR (Propofol MCT)) OR (Propofol Rovi)) OR (Propofol-Lipuro)) OR (Recofol)) OR (Aquafol)) OR (Propofol Abbott)) AND ((((((((((“Autophagy” [Mesh]) OR (Autophagy, Cellular)) OR (Cellular Autophagy)) OR (Autophagocytosis)) OR (Reticulophagy)) OR (ER-Phagy)) OR (ER Phagy)) OR (Nucleophagy)) OR (Ribophagy)) OR (Lipophagy)]. A data collection table was made for the key data from included studies, including the name of the first author, publication year, experimental model, types of injury, the status of autophagy, associated genes or pathways, and the main findings of the included studies. Finally, 21 studies were included. Among these, ten, five, and six eligible studies explored the effects of propofol on the nervous system and the roles of autophagy from neuronal cell damage *in vitro*, nervous system injury of animal models, and cognitive dysfunction, respectively.

### Neuronal cells damage *in vitro*

Ten publications reported that autophagy could involve the action of the effect of propofol in nervous system through the neurocyte tests *in vitro*. The neurocyte used in these experimental models included mouse neuron cells (HT22 cells), human umbilical vein endothelial cells (HUVECs), ReNcell CX cells, primary rat cerebral cortical neurons, mouse embryonic fibroblast, rat pheochromocytoma cells (PC12), and human neuroblastoma (SH-SY5Y cell). Cells were treated with diffident concentrations of propofol (1, 5, 20, 50, 100, 200, 300, 400, and 500 μM, 25, 50, 100, and 150 μmol/L, 2, 5, 10, 20, and 50 mg/mL). Three publications reported the neurotoxicity of propofol via inducing autophagy and five publications reported neuroprotective properties of propofol via inhibiting autophagy. Two publications report the dual effects of propofol on the nervous system, a clinically relevant dose of propofol inhibits excessive autophagy and promote cell proliferation, while high-dose propofol produces neurotoxicity via inducing autophagy. They have demonstrated that multiple genes and signaling pathways have been involved in regulating autophagy.

The relevant information and the main findings extracted from ten relevant studies reporting neuronal cell damage *in vitro* are summarized in Table 1.

**Table 1:** Neuronal cells damage *in vitro*.

Study/reference	Experimental model	Types of neuropathy	Status of autophagy	Associated genes or pathways	Main findings
Liu et al. (2019)	Mouse neuron cell (HT22 cells)	Neuronal cells injury	Activation	Induced the expression of LC3-I and LC3-II via the REST/BDNF signaling pathway	Propofol inhibited cell proliferation and promoted apoptosis in a dose-dependent manner by promoting the expression of LC3-I, LC3-II. The overexpression of REST reduced the damage of propofol to mouse neuronal cells, which might inhibit the apoptosis by inhibiting autophagy of neuronal cells
Qiao et al. (2017)	ReNcell CX cells	Cell damage	Activation or inhibited	calcium-mediated autophagy pathway	A high concentration of propofol (200 μM) induced the overactivation of InsP3Rs and RyRs through Ca^2+^-mediated pathway to cause excessive autophagy, which was harmful to NPC cells. However, clinically relevant doses of propofol could promote the proliferation of NPC cells and elevated the differentiation of neurons
Zhang et al. (2021)	Mouse hippocampal neuron HT22	Cell injury	Activation	Up-regulating of Beclin1and LC3 and down-regulating of p62	Propofol induced HT22 cell injury via autophagy activation by up-regulating of Beclin1and LC3 and down-regulating of p62. NAPS4 overexpression inhibited propofol-induced autophagy and improved HT22 cell damage
Xiu et al. (2022)	Human neuroblastoma SH-SY5Y cell	Cell injury	Activation	Downregulated miR-17-5p expression	Propofol led to neurotoxicity in SH-SY5Y cells by up-regulating of Atg5, Beclin1, and LC3II/I and down-regulating of p62. Propofol treatment down-regulated miR-17-5p expression. MiR-17-5p alleviated PPF induced cell damage by inhibiting BCL2L11
Chen et al. (2013)	HUVECs	H/R injury	Inhibited	Down-regulating apoptotic protein Bax, up-regulating anti-apoptotic protein Bcl-2	Post-conditioning with Propofol after hypoxia treatment can effectively inhibit H/R induced apoptosis and autophagy in HUVECs by inhibiting PARP cleavage, up regulating Bcl-2, down regulating Bax and preventing the transformation from LC3-I to LC3-II. And propofol posthypoxia treatment in H/R induces different miRNA expression patterns
Sun et al. (2018)	Primary rat cerebral cortical neurons	Ischemia-reperfusion injury	Inhibited	Ca^2+^/CaMKKβ/AMPK/mTOR pathway	Propofol dose-dependently reduced the autophagy level induced by OGD/R via down-regulating the expression of Beclin-1 and LC3-II/I and up-regulating the expression of p62. Propofol significantly reduced the intracellular Ca^2+^ concentration caused by OGD/R. Propofol improved the neuronal damage triggered by OGD/R by inhibiting autophagy by Ca^2+^/CaMKKβ/AMPK/mTOR pathway
Xu et al. (2020)	Mouse embryonic fibroblast	Cell damage	Activation or inhibited	ATG5-and intracellular Ca^2+^ channels	Dose-dependent propofol affected the proliferation and death of fibroblasts via regulation of autophagy by affecting calcium release. AGT5 played an important role in this process
Yang et al. (2019)	Rat pheochromocytoma cells (PC12)	Cell damage (Alzheimer’s disease)	Inhibited	Over-activating InsP3Rs/RYRs	Propofol led to insufficient autophagic flux and cell damage in AD cells via the induction of calcium imbalance by over-activating InsP3Rs and/or RYRs. Either RYR or InsP3R antagonists could improve cell death, but not the combination of both
Zhou et al. (2021)	Rat pheochromocytoma cells (PC12)	Cobalt chloride (CoCl_2_)-induced injury	Inhibited	Down-regulating miR-134	Propofol improved CoCl_2_-induced injury in rat pheochromocytoma cells via inhibiting autophagy, apoptosis, and oxidative stress by down-regulating miR-134
Zhang et al. (2022)	Mouse hippocampal neurons	TNF-α-mediated p-tau deposition	Inhibited	p62/Keap1/Nrf2 pathway	Propofol reduced neuronal damage caused by TNF-α-mediated p-Tau deposition. Knocking down p62, over expressing Keap1, or inhibiting Nrf2 through the p62/Keap1/Nrf2 pathway might played roles in this action

### Autophagy-associated proteins (Beclin1, Bcl-2 LC3-I, LC3-II and p62)

Effects of propofol on the nervous system may be closely related to autophagy-related proteins such as Beclin1, Bcl-2, LC3-I, LC3-II and p62. Beclin1 is the first mammalian autophagy-related gene that was discovered by the Levine group in 1999, it can induce autophagy and inhibit tumorigenesis (Liang et al., 1999). Bcl-2 is one of the important factors in the antiapoptotic family members. Previous studies have shown that Bcl-2 inhibits Beclin 1-dependent autophagy (Pattingre and Levine, 2006). LC3 widely exists in mammalian tissues. During autophagy, cytoplasmic components are engulfed by autophagosomes, while LC3-I is conjugated to phosphatidylethanolamine to form LC3-II (Tanida et al., 2008). It was reported that pre-treatment with propofol significantly decreased the hypoxia-induced accumulation of LC3-II, indicating propofol could reduce hypoxia-induced autophagic cell death (Ning et al., 2017). P62 (SQSTM1) had been demonstrated that it was associated with neurodegenerative disease by inducing autophagy and mitophagy dysfunction (Liu et al., 2017). Beclin1, Bcl-2, LC3-I, and LC3-II are positively correlated with autophagy, and p62 is negatively correlated with autophagy. Liu et al. (2019) demonstrated that propofol disrupted the function of the nervous system via the promotion of apoptosis and autophagy by promoting the expression of LC3-I and LC3-II. Similarly, Zhang et al. (2021) showed that propofol causes HT22 cell injury via inducing autophagy by up-regulating Beclin1 and LC3 and down-regulating of p62. At the same time, they found different ways to improve the neurotoxicity of propofol through experiments. Liu et al. (2019) found that the over-expression of REST reduces the damage of propofol on mouse neuronal cells via inhibition of autophagy through the REST/BDNF signaling pathway. Zhang et al. (2021) reported that NPAS4 could improve neurotoxicity by inhibiting autophagy induced by propofol. Conversely, Chen et al. (2013) demonstrated that post-conditioning with propofol after hypoxia treatment can effectively inhibit H/R-induced apoptosis and autophagy in HUVECs by inhibiting PARP cleavage, up-regulating Bcl-2, down-regulating Bax, and preventing the transformation from LC3-I to LC3-II. Zhang et al. (2022) also proved the neuroprotective properties of propofol. They found that propofol reduces neuronal damage caused by TNF-α-mediated p-tau deposition via knocking down p62, over-expressing Keap1, or inhibiting Nrf2 through p62/Keap1/Nrf2 pathway. The above studies suggest that propofol exerts its biological function on the nervous system by regulating multiple autophagy-related proteins.

### Ca^2*+*^ regulates autophagy

Calcium (Ca^2+^), as a second messenger, is a crucial regulator of many processes (Smaili et al., 2013). Similarly, intracellular calcium plays an important role in autophagy and apoptosis (Hoyer-Hansen et al., 2007; Cardenas et al., 2010). Several Ca^2+^ channels and pathways are involved in autophagy regulation, such as IP3 receptors, and Ca^2+^/CaMKKβ/AMPK/mTOR pathway. Qiao et al. (2017) reported that a high concentration of propofol (200 μM) caused the overactivation of InsP3Rs and RyRs through Ca^2+^-mediated pathway to cause excessive autophagy, which is harmful to NPC cells *in vitro*. However, clinically relevant doses of propofol can promote the proliferation of NPC cells and increase the differentiation of neurons. Similar to the findings reported Qiao et al. (2017) and Xu et al. (2020) demonstrated that dose-dependent propofol affects the proliferation and death of mouse fibroblasts *in vitro* via regulation of autophagy by affecting calcium release, and AGT5 plays an important role in this process. More importantly, the cells with Alzheimer’s disease characteristics were more susceptible to propofol neurotoxicity (Xie et al., 2007). Yang et al. (2019) reported that propofol causes insufficient autophagic flux and cell damage in AD cells via the induction of calcium imbalance by over-activating InsP3Rs and/or RYRs. Either RYR or InsP3R antagonists can improve cell death, but not the combination of both. On the contrary, Sun et al. (2018) discovered the neuroprotective effect of propofol in the I/R injury model induced by OGD/R. This study showed that propofol dose-dependently reduces the autophagy level induced by OGD/R via down-regulating the expression of Beclin-1 and LC3-II/I and up-regulating the expression of p62. Moreover, propofol significantly reduces the intracellular Ca^2+^ concentration induced by OGD/R. Thus, propofol improves neuronal damage triggered by OGD/R via inhibition of autophagy by the Ca^2+^/CaMKKβ/AMPK/mTOR pathway. Calpain is a calcium-dependent protease that plays an important role in neuronal autophagy caused by inflammation. Propofol has been found to suppress hippocampal neuron autophagy by regulating Calpain and calcium-dependent signaling pathway (Li et al., 2020).

### MicroRNAs

MicroRNAs (miRNAs), as endogenous non-coding small RNAs, regulate gene expression at the posttranslational level and have many important biological effects, such as cell differentiation, cell replication, autophagy, and apoptosis (Alvarez-Garcia and Miska, 2005; Kim, 2005; Kloosterman and Plasterk, 2006; Landgraf et al., 2007). A large body of evidence shows that a close relationship exists between miRNA and autophagy in nervous system diseases (Eyileten et al., 2021; Xu et al., 2021; Yu et al., 2021). Specific miRNAs have been found to control the biogenesis of autophagosomes, involving in the degeneration of medium spiny neurons by regulating autophagy (Oh et al., 2023). Interestingly, many investigators have implied that there is a positive association between propofol treatment and the altered expression of miRNAs or the status of autophagy (Han et al., 2021; Xiao et al., 2021). Consequently, miRNAs may also play a significant role in the influence of propofol on the nervous system. Xiu et al. (2022) found that propofol causes neurotoxicity in SH-SY5Y cells by up-regulating Atg5, Beclin1, and LC3II/I and down-regulating of p62. Meanwhile, propofol treatment down-regulates miR-17-5p expression. MiR-17-5p alleviated PPF-induced cell damage by inhibiting BCL2L11. Conversely, Zhou et al. (2021) reported that propofol improves CoCl_2_-induced injury in rat pheochromocytoma cells (PC12) via inhibition of autophagy, apoptosis, and oxidative stress by down-regulating miR-134. The aforementioned studies demonstrated that several miRNAs (i.e., miR-17-5p and miR-134) involved in the actions of propofol-related neuropathy by regulating cellular autophagy.

### Nervous system injury of animal model

Five publications reported the neuroprotective properties of propofol in nervous system injury of animal models and the key roles of autophagy. The experimental models in these trials included rats and mice, and the types of neuropathy included electroconvulsive therapy injury, cerebral ischemia-reperfusion injury, and acute ischemic stroke. Most (80%) of the included studies reported that propofol protected damaged neurons via inhibition of autophagy through genes or pathways.

The relevant information and the main findings extracted from five relevant studies reporting neuronal cell damage *in vitro* are summarized in Table 2.

**Table 2:** Nervous system injury of animal model.

Study/reference	Experimental model	Types of neuropathy	Status of autophagy	Associated genes or pathways	Main findings
Cui et al. (2013)	Rat	Cerebral ischemia-reperfusion injury	Inhibited	NF-κB/p53 signaling pathway	Propofol of relevant concentration prevented autophagy and apoptosis in hippocampus of rats after brain I/R injury by up regulating Bcl-2 protein expression, down regulating Beclin 1 protein expression, PUMA protein level, Bax protein expression, and LC3-II /LC3-I ratio through the NF-κB/p53 signaling pathway
Li et al. (2016)	Rat	Electroconvulsive therapy injury	Inhibited	Down-regulating the expression of Beclin-1 and LC3-II/I	Propofol could prevent depressed rats against ECS induced autophagy by down-regulating the expression of Beclin-1 and LC3-II/I, thus improving their learning and memory impairment
Wang et al. (2020)	Mouse	Acute ischemic stroke	Inhibited	mTOR/S6K1 pathway	Propofol treatment improved the prognosis of acute ischemic stroke via reduction of α-synuclein aggregation and inhibition of stroke-induced autophagy which caused by the activation of the mTOR/S6K1 signaling pathway
Sun et al. (2021)	Mouse	Ischemia/reperfusion injury	Inhibited	Down-regulating lncRNA SNHG14	SNHG14 aggravated CI/R injury via activation of autophagy by up-regulating Atg5 and Beclin 1. Pprpofol improved I/R injury via inhibition of lncRNA SNHG14 by the p38 MAPK signaling pathway
Chen and Li (2021)	Rat	I/R injury	Activation	PI3K/Akt pathway	Propofol preconditioning improved the neural function of rats with I/R injury by inhibiting oxidative stress response and proinflammatory factor secretion. Propofol also promoted autophagy in cerebral I/R injury through PI3K/Akt pathway to protect the brain tissue of rats with I/R injury

### Cerebral ischemia-reperfusion injury and acute ischemic stroke

As a common cerebrovascular disease, cerebral ischemia seriously affects the health and quality of life of middle-aged and elderly people (Diaz-Canestro et al., 2018). Ischemic stroke is caused by the occlusion of the arteries supplying cerebral blood for various reasons, which leads to a decrease in blood flow in brain tissue. It is more extensive cerebral ischemia, which is also a cerebral ischemic disease in essence. During the rescue and treatment of ischemic diseases, the main factor causing tissue damage is caused by excessive free radicals attacking cells in this part of the tissue that has regained blood supply after the blood supply is restored, to cause more serious brain function damage (Jayaraj et al., 2019). Propofol has been proved to exert a protective effect on organs with ischemia/reperfusion injury by regulating autophagic flux (X. Liu et al., 2022). Studies have shown that propofol therapy attenuates cerebral ischemia-reperfusion injury by inhibiting endoplasmic reticulum stress and using heme oxygenase-1 in rat models of cerebral I/R injury (Liang et al., 2013; Xu et al., 2018). Autophagy also played an important role through different genes or pathways, which was confirmed by four published articles. Cui et al. (2013) confirmed by rats test that propofol of relevant concentration prevents autophagy and apoptosis in the hippocampus of rats after brain I/R injury by up-regulating Bcl-2 protein expression, down-regulating Beclin 1 protein expression, PUMA protein level, Bax protein expression, and LC3-II /LC3-I ratio through NF-κB/p53 signaling pathway. Sun et al. (2021) also found that propofol improves CI/R injury via inhibition of lncRNA SNHG14 by the p38 MAPK signaling pathway because of SNHG14 aggravating CI/R injury via activation of autophagy by up-regulating Atg5 and Beclin 1. Wang et al. (2020) demonstrated that propofol treatment improves the prognosis of acute ischemic stroke via reduction of α-synuclein aggregation and inhibition of stroke-induced autophagy which was caused by the activation of the mTOR/ S6K1 signaling pathway. These experiments on rats and mice all have confirmed that propofol plays a neuroprotective role by inhibiting autophagy. On the contrary, Chen and Li (2021) demonstrated that propofol promotes autophagy in cerebral I/R injury through PI3K/Akt pathway to protect the brain tissue of rats with I/R injury. In addition, propofol preconditioning improves the neural function of rats with I/R injury by inhibiting oxidative stress response and proinflammatory factor secretion. The above evidence indicated that propofol could prevent cerebral ischemia-reperfusion injury or acute ischemic stroke by inhibiting autophagy.

### Electroconvulsive therapy injury

Electroshock therapy (ECT) is a method to treat depression by using a certain amount of current to pass through the brain to cause loss of consciousness and convulsions, to achieve the purpose of treatment (Pagnin et al., 2004) However, ECT has some adverse reactions, such as headaches, nausea and vomiting, transient degenerative memory loss, and the impairment of learning and memory (Pigot et al., 2008; Li et al., 2012). ECT has been found to be associated with neurotoxic injury (Zhong et al., 2021). Recent studies have found that ECT may cause nervous system damage by inducing autophagy, and propofol can affect the nervous system by autophagy (Otabe et al., 2014). Thereby, Li et al. (2016) designed this test, they demonstrated that propofol prevents depressed rats against electroconvulsive shock (ECS)-induced autophagy by down-regulating the expression of Beclin-1 and LC3-II/I, then improving their learning and memory impairment. However, due to the lack of relevant studies, more and further research is needed.

### Cognitive dysfunction

Postoperative cognitive dysfunction (POCD) is an important complication of anesthesia and surgery, which can cause short-term cognitive impairment and memory loss (Shoair et al., 2015; Vutskits and Xie, 2016). Long-term sleep disorder is also one of the causes of cognitive dysfunction (Dzierzewski et al., 2018). Autophagy has been proposed to be correlated to the development of cognitive dysfunction. Some specific interventions exert the neuroprotection by restoring neuronal tau homeostasis by enhancing cellular autophagy (Zhang et al., 2023). Propofol, as the most commonly used intravenous anesthetic in clinical anesthesia and one of the treatment schemes for sleep disorders, is necessary to study the relationship with cognitive dysfunction (Rabelo et al., 2013). Six publications reported the relationship between propofol and cognitive dysfunction and the key roles of autophagy. The relevant information and the main findings extracted from 10 relevant studies reporting neuronal cells damage *in vitro* are summarized in Table 3.

**Table 3:** Studies reported with cognitive dysfunction.

Study/reference	Experimental model	Types of neuropathy	Status of autophagy	Associated genes or pathways	Main findings
Yang et al. (2017)	Aged rats	Post-operative cognitive dysfunction	Inhibited	Down-regulating the expression of Beclin-1 and LC3-II/I up-regulating the expression of p62	Prolonged exposure to propofol induced cognitive dysfunction via inhibition of autophagy in hippocampus of aged rats by down-regulating the expression of Beclin-1 and LC3-II/I and up-regulating the expression of p62. DDS could up-regulate autophagy to protect against propofol induced POCD
Cho et al. (2018)	GFP-LC3 adult mice	Post-operative cognitive dysfunction	Activation	NMDAR dependent signaling pathways	Propofol caused transient cognitive dysfunction via the changing of NMDAR dependent signaling pathways by down-regulation of p-CAMKIIα and PSD-95 levels, but propofol could restore their cognitive function via the enhancement of the autophagic flux and autophagic clearance rate by up-regulating the activities of cathepsin D and LAMP2
Logan et al. (2018)	7 days-old mice	Acute neurotoxicity	Activation	lncRNA profile and the associated signaling pathways	When the 7 days-old mice were exposed to propofol, the caspase-3 enzyme activity in their hippocampus increased, which caused apoptosis and autophagy of the hippocampal neurons. The possible mechanism was that propofol altered Long Non-coding RNA profiles
Li et al. (2020)	Mouse hippocampal neurons	Post-operative cognitive dysfunction	Inhibited	Calcium and calcium-dependent signalling pathway	Propofol concentration-dependently significantly inhibited TNF-α-induced autophagy of hippocampal neurons via NMDA receptor. This mechanism might be associated with calcium and calcium dependent signaling pathways, specially CAMK II and calpain-2
Yang et al. (2020)	Aged rats	Post-operative cognitive dysfunction	Inhibited	Down-regulating the expression of Beclin-1 and LC3B, increased α-synuclein oligomerization	Propofol alone and propofol/surgery resulted in postoperative cognitive dysfunction via the inhibition of autophagy in hippocampus (by down-regulating the expression of Beclin-1 and LC3B) and increased the α-synuclein oligomerization. Rapamycin preconditioning reversed these effects by promoting autophagy
Dai et al. (2021)	Adult male rats	Sleep deprivation-induced learning and memory impairment	Inhibited	Down-regulating of Beclin1, PINK1, parkin, p62, and LC3	Propofol improved the learning and memory impairment in rats via inhibition of excessive autophagy and mitophagy in hippocampal neurons by down-regulating of Beclin1, PINK1, parkin, p62, and LC3

Four related publications have reported that propofol leads to can cognitive dysfunction via inhibition or activation of autophagy through different pathways whether in adult rats or newborn mice. Two of the included studies reported the status of autophagy was inhibition. Yang et al. (2017) found that prolonged exposure to propofol causes cognitive dysfunction via inhibition of autophagy in the hippocampus of aged rats by down-regulating the expression of Beclin-1 and LC3-II/I and up-regulating the expression of p62. But Logan et al. (2018) reported that propofol reduces the cognitive function of 7 days mice via activation apoptosis and autophagy by increasing the activity of caspase 3 enzyme in their hippocampus through altering long non-coding RNA profiles. Otherwise, although Cho et al. (2018) found that propofol leads to transient cognitive dysfunction via the changing of NMDAR-dependent signaling pathways by down-regulation of p-CAMKIIα and PSD-95 levels. They also reported that propofol restores their cognitive function via the enhancement of the autophagic flux and autophagic clearance rate by up-regulating the activities of cathepsin D and LAMP2.

The other two studies showed the opposite effect of propofol. Li et al. (2020) reported that Propofol improves cognitive dysfunction because of its concentration-dependently inhibition of TNF-α-induced autophagy of hippocampal neurons via NMDA receptor. This mechanism may be related to calcium and calcium-dependent signaling pathways, specially CAMK II and calpain-2. Dai et al. (2021) demonstrated that propofol improves learning and memory impairment in sleep deprivation (SD) rats via inhibition of excessive autophagy and mitophagy in hippocampal neurons by down-regulating of Beclin1, PINK1, parkin, p62, and LC3. Taken together, most of the relevant included studies demonstrate that the improvement of cognitive dysfunction exerting by propofol may be associated with the inhibition of autophagy which characterized by suppressing the autophagy related proteins and the interactions with some specific signal cascades.

**Figure 1:**
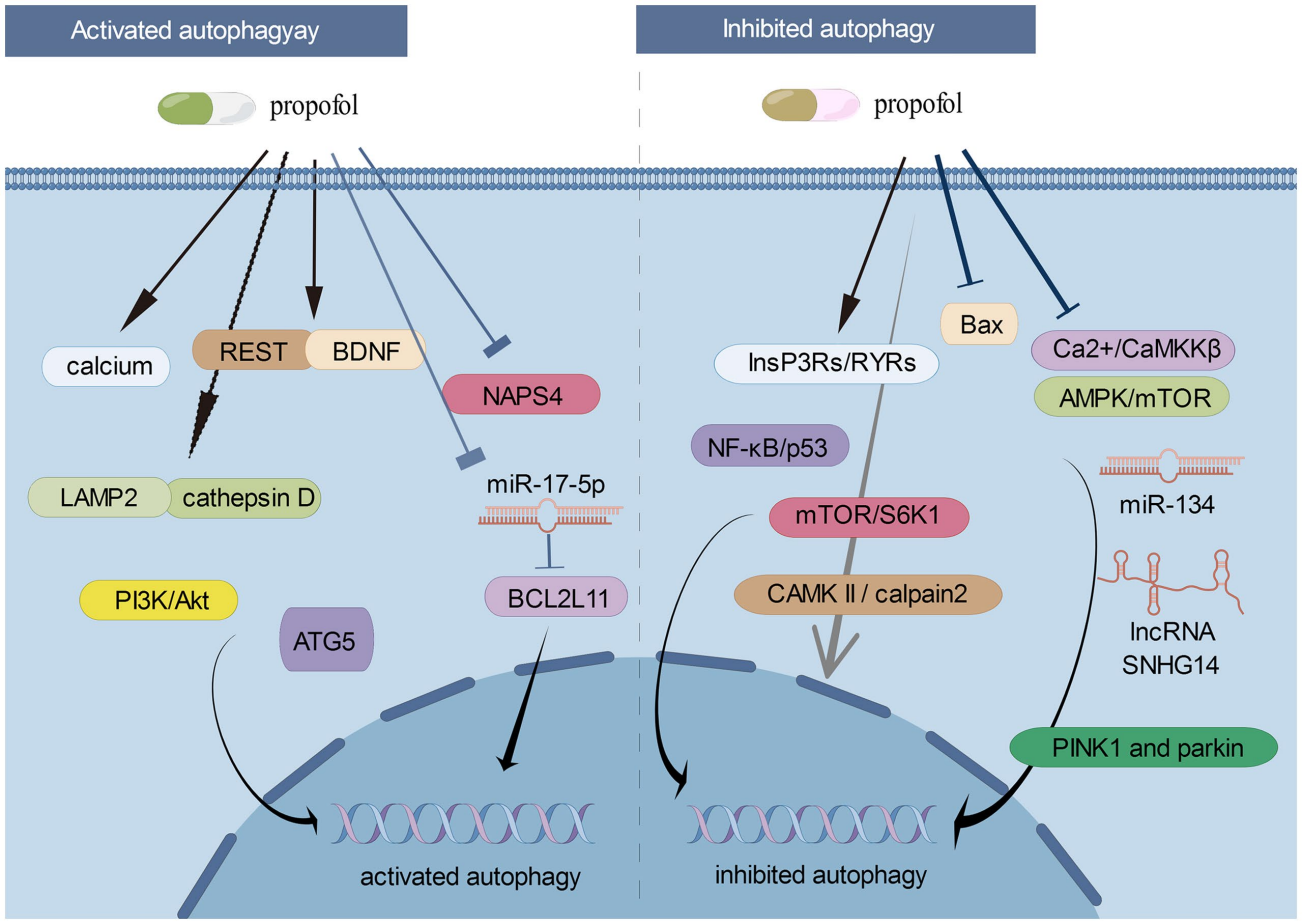
The roles of activated/inhibited autophagy and the corresponding signaling cascades in propofol-related neurological diseases (By Figdraw).

Figure 1 showed the roles of activated/inhibited autophagy and the corresponding signaling cascades in propofol-related neurological diseases.

#### Propofol-associated side effects

Adverse neurological effects of propofol are relatively rare, but may occur under certain circumstances. In some cases, propofol may cause neuroexcitatory responses such as muscle tremors, twitching, or involuntary movements. Additionally, it may also have some detrimental effect on memory and concentration. Under the condition of rapidly administration or at a high dose, propofol may cause excessive depth of sedation, leading to a coma. Some patients received propofol may experience headaches or other altered states of consciousness. The present review highlighted the potential involvement of the autophagy in the propofol-induced neuroprotective effect. However, the available included studies did not examine whether these side effects or neurotoxicity induced by propofol were related to the alterations of autophagic flux and the expressions of autophagy related proteins, which needs to be further verified by additional studies.

#### Autophagy-mediated pediatric neurological disorders under propofol treatment

Propofol is one of the most commonly used intravenous general anesthetics in clinical anesthesia, and it is also used in general anesthesia for infants and children (Zhang and Li, 2023). Nevertheless, some clinical and preclinical studies have found that propofol causes damage to the immature nervous system, which may lead to neurodevelopmental disorders and cognitive dysfunction in children. An *in vitro* study demonstrated that low concentration of propofol improved the survival rate of neurons, while high concentration of propofol promoted the cell apoptosis and decreased the cell viability. A clinical study (Koch et al., 2018) reported that propofol induction of anaesthesia in children triggers epileptiform discharges, whereas to a lesser extent than sevoflurane or remifentanil does. This study indicates that propofol has a bad impact on postoperative brain function but it is relatively safe when compare to sevoflurane and higher level of remifentanil for anaesthesia induction in children. A previous clinical study (Sheridan et al., 2012) also demonstrated that propofol was effective for the abortive treatment of pediatric migraine headache. At present, the potential mechanism of the action of propofol-mediated neurological impacts has not been fully elucidated due to relevant experimental studies were limited in literatures. Few *in vivo* studies have investigated the neurological effect of propofol by applying a juvenile animal model. Therefore, the biological functioning of autophagy in propofol-treated neurological diseases are still warranted to explore in the future.

#### Undiscovered role of microbiome in propofol-treated neurological diseases

Microbiome has been found to exhibit some important impact on the nervous system, forming the theoretical framework of the microbiota-gut-brain axis (Chaudhry et al., 2023). Microbiome involves in the development of multiple neurological diseases via its diverse biological functions, including neurotransmitter regulation, immunomodulation, neurodevelopment and plasticity, modulation of inflammation, and neurohormonal and metabolic regulation. Enteric microbiomes play roles in neuroimmune interactions through the phylogeny of neuroimmunity, enteric neuronal and glial regulation of immunity (Margolis and Gershon, 2016). Autophagy has been found to involve in the gut microbial metabolites and neuroinflammation (Li et al., 2023). Intriguingly, previous study demonstrates that propofol exerts an impact on intestinal microbiome and metabolome (H. Liu et al., 2022). Within the scope of this review, propofol showing the neuroprotective effects may be associated with the modulation of cellular autophagy, while microbiomes may play a pivotal role in this action. However, the potential relationship among propofol, microbiota-gut-brain, and autophagy has yet to be elucidated, which is urgently needed to confirm by further studies.

#### Propofol in neuroautoimmune diseases, ASD, and ADHD

Propofol can alter immune cell functions and have certain immunosuppressive properties, which could potentially be beneficial in managing autoimmune conditions (Shiratsuchi et al., 2009). A previous study showed that propofol hemisuccinate could suppress autoimmune encephalomyelitis, suggesting its curative effect on acute exacerbations of multiple sclerosis (Vansant et al., 2009). Some case reports also indicated that propofol had certain effects on the treatment of multiple sclerosis (Ohshita et al., 2017). However, the underlying mechanisms are poorly investigated. The exact roles of autophagy in the propofol-treated neuroautoimmune diseases are still warranted to be explored in the future.

Patients with autism spectrum disorders (ASD) and attention-deficit hyperactivity disorder (ADHD) have a high incidence of neurologic comorbidities. Some studies have explored the effects of propofol on anesthesia, sedation, social behavior in individuals with ASD and ADHD (Spiller et al., 2013; Abulebda et al., 2018; Walsh et al., 2018). However, the field of propofol and ASD or ADHD is still in its early stages, and more research is needed to understand the potential benefits, risks, and optimal methods of administration. On the other hand, the experimental data related to propofol-associated ASD and ADHD are also limited. Therefore, the role of autophagy that underlies the effect of propofol in ASD and ADHD remains unknown.

#### Neurotransmitter receptors in actions of propofol

The action of propofol involves a positive modulation of the inhibitory function of the neurotransmitter g-aminobutyric acid through GABA receptors. Meanwhile, other receptors, such as glycine receptor, nicotinic receptor and M1 muscarinic receptor have been proposed to play roles in propofol-mediated pleiotropic effects. Propofol can activate both GABA_A_ and glycine receptors in spinal cord. Besides, propofol also depresses cerebellar Purkinje cell activity by activating GABA_A_ and glycine receptors (Jin et al., 2015). It was reported that halogenation of propofol more mightily affected the modulation of homomeric glycine receptors than that of α1β3γ2L GABA_A_ receptors (Germann et al., 2016). In addition to GABA_A_ and glycine receptors, propofol also has an inhibitory effect on serotonin receptors and a facilitatory action on N-methyl-D-aspartate (NMDA) in the central nervous system (Sweni et al., 2011). Westphalen and Hemmings (2003) demonstrated that propofol at clinical concentrations did not affect excitatory glutamatergic transmission on presynaptic neuronal transporters. Based on the above evidence, propofol can interact with different neurotransmitter receptors, including GABA, glycine, serotonin, and NMDA receptors. However, after reviewing the included studies and other available relevant studies, most of them investigated that propofol exerted the neurologic effects by modulating autophagy via the interaction with GABA receptors. Therefore, the interaction between autophagy and other neurotransmitter receptors in propofol-treated neurological diseases will require additional research.

#### The possible counterparts of autophagy and phagocytosis

Both phagocytosis and autophagy are intracellular degradation processes, maintaining cellular function and physiological homeostasis. Some autophagic processes may involve phagocytosis of extracellular particles. However, phagocytosis and autophagy remain distinct biological processes. Phagocytosis primarily involves phagocytosis and degradation of external microorganisms, cellular debris, extracellular aggregates, and other large particles or microparticles, while autophagy is primarily an intracellular self-degradation process. Phagocytosis enables cells to encapsulate and engulf external particles to form phagosome, while autophagy is primarily achieved through the formation of intracellular membrane structures (i.e., autophagosome). Phagocytosis is a part of the immune system used to clear infected microorganisms and maintain tissue homeostasis, while autophagy is extensively involved in multiple physiological processes, such as intracellular protein-quality control, organelle maintenance, metabolic adaptation, and cellular stress. Currently, two studies have explored the role of phagocytosis in propofol-mediated neurological alterations. Propofol can stimulate microglial phagocytosis at ambient pressure, while elevated pressure may reduce phagocytosis (Yu et al., 2011). Propofol suppresses microglial phagocytosis, thus inhibiting microglial activity (Cai et al., 2021). Due to limited relevant studies, the possible counterparts of autophagy and phagocytosis in propofol-treated neurological diseases are still unclear, which is expecting a future study.

## Conclusion

This review highlights the effect of propofol on the nervous system and the crucial roles of autophagy, and we can find that propofol is a double-edged sword. In the included studies, some of them reported that propofol causes neuronal cell damage by regulating autophagy, leading to cognitive dysfunction and other neurological diseases, especially high concentrations and doses of propofol. However, some of them have shown that in the model of existing nervous system diseases, such as cerebral ischemia-reperfusion injury, electroconvulsive therapy injury, cobalt chloride (CoCl_2_)-induced injury, TNF-α-induced injury and sleep deprivation-induced injury, propofol plays a neuroprotective role by regulating autophagy, and improves the degree of nerve damage. Autophagy plays a crucial role by regulating oxidative stress, inflammatory response, calcium release, and other mechanisms, which may be related to the interaction of a variety of related proteins and signal cascades. With extensive in-depth research in the future, the autophagy mechanism mediated by propofol will be fully understood, so as to better apply the neuroprotective effect of propofol in clinical work and minimize the neurotoxicity of propofol.

## Author contributions

SY: Investigation, Writing – original draft, Writing – review & editing. JL: Conceptualization, Data curation, Writing – review & editing. XL: Methodology, Writing – original draft. YL: Formal analysis, Funding acquisition, Writing – review & editing. GL: Writing – original draft, Writing – review & editing.

## References

[ref1] Abulebda K. Louer R. Lutfi R. Ahmed S. S. (2018). A comparison of safety and efficacy of dexmedetomidine and propofol in children with autism and autism spectrum disorders undergoing magnetic resonance imaging. J. Autism Dev. Disord. 48, 3127–3132. doi: 10.1007/s10803-018-3582-1, PMID: 29680960

[ref2] Alvarez-Garcia I. Miska E. A. (2005). MicroRNA functions in animal development and human disease. Development 132, 4653–4662. doi: 10.1242/dev.02073 16224045

[ref3] Bosnjak Z. J. Logan S. Liu Y. Bai X. (2016). Recent insights into molecular mechanisms of propofol-induced developmental neurotoxicity: implications for the protective strategies. Anesth. Analg. 123, 1286–1296. doi: 10.1213/ANE.0000000000001544, PMID: 27551735 PMC5073000

[ref4] Cai X. Li Y. Zheng X. Hu R. Li Y. Xiao L.. (2021). Propofol suppresses microglial phagocytosis through the downregulation of MFG-E8. J. Neuroinflammation 18:18. doi: 10.1186/s12974-020-02061-3, PMID: 33422097 PMC7796553

[ref5] Cardenas C. Miller R. A. Smith I. Bui T. Molgo J. Muller M.. (2010). Essential regulation of cell bioenergetics by constitutive insp3 receptor Ca^2+^ transfer to mitochondria. Cells 142, 270–283. doi: 10.1016/j.cell.2010.06.007, PMID: 20655468 PMC2911450

[ref6] Chaudhry T. S. Senapati S. G. Gadam S. Mannam H. P. S. S. Voruganti H. V. Abbasi Z.. (2023). The impact of microbiota on the gut-brain axis: examining the complex interplay and implications. J. Clin. Med. 12:5231. doi: 10.3390/jcm12165231, PMID: 37629273 PMC10455396

[ref7] Chen W. W. Zhang X. Huang W. J. (2016). Role of neuroinflammation in neurodegenerative diseases (review). Mol. Med. Rep. 13, 3391–3396. doi: 10.3892/mmr.2016.4948 26935478 PMC4805095

[ref8] Chen X. H. Zhou X. Yang X. Y. Zhou Z. B. Lu D. H. Tang Y.. (2016). Propofol protects against H_2_O_2_-induced oxidative injury in differentiated pc12 cells via inhibition of Ca^2+^-dependent NADPH oxidase. Cell. Mol. Neurobiol. 36, 541–551. doi: 10.1007/s10571-015-0235-1, PMID: 26162968 PMC11482435

[ref9] Chen Y. Li Z. (2021). Protective effects of propofol on rats with cerebral ischemia-reperfusion injury via the PI3K/Akt pathway. J. Mol. Neurosci. 71, 810–820. doi: 10.1007/s12031-020-01703-8, PMID: 32984935

[ref10] Chen Z. Hu Z. Lu Z. Cai S. Gu X. Zhuang H.. (2013). Differential microRNA profiling in a cellular hypoxia reoxygenation model upon posthypoxic propofol treatment reveals alterations in autophagy signaling network. Oxidative Med. Cell. Longev. 2013:378484, 1–11. doi: 10.1155/2013/378484 PMC3885199 24454982

[ref11] Cho S. Jung Y. J. Suh E. C. Baik H. J. Han J. I. Lee G. Y.. (2018). The recovery from transient cognitive dysfunction induced by propofol was associated with enhanced autophagic flux in normal healthy adult mice. Brain Res. 1700, 99–108. doi: 10.1016/j.brainres.2018.07.007 30006294

[ref12] Collins G. G. (1988). Effects of the anaesthetic 2,6-diisopropylphenol on synaptic transmission in the rat olfactory cortex slice. Br. J. Pharmacol. 95, 939–949. doi: 10.1111/j.1476-5381.1988.tb11724.x, PMID: 2850066 PMC1854202

[ref13] Cui D. R. Wang L. Jiang W. Qi A. H. Zhou Q. H. Zhang X. L. (2013). Propofol prevents cerebral ischemia-triggered autophagy activation and cell death in the rat hippocampus through the NF-κB/p53 signaling pathway. Neuroscience 246, 117–132. doi: 10.1016/j.neuroscience.2013.04.054, PMID: 23644056

[ref14] Cui D. Wang L. Qi A. Zhou Q. Zhang X. Jiang W. (2012). Propofol prevents autophagic cell death following oxygen and glucose deprivation in pc12 cells and cerebral ischemia-reperfusion injury in rats. PLoS One 7:e35324. doi: 10.1371/journal.pone.0035324, PMID: 22509406 PMC3324553

[ref15] Dai W. Xiao Y. Tu Y. Xiao F. Lu Y. Qin Y.. (2021). Propofol protects hippocampal neurons in sleep-deprived rats by inhibiting mitophagy and autophagy. Ann. Transl. Med. 9:1427. doi: 10.21037/atm-21-3872, PMID: 34733979 PMC8506745

[ref16] Diaz-Canestro C. Merlini M. Bonetti N. R. Liberale L. Wust P. Briand-Schumacher S.. (2018). Sirtuin 5 as a novel target to blunt blood-brain barrier damage induced by cerebral ischemia/reperfusion injury. Int. J. Cardiol. 260, 148–155. doi: 10.1016/j.ijcard.2017.12.060, PMID: 29622432

[ref17] Dzierzewski J. M. Dautovich N. Ravyts S. (2018). Sleep and cognition in older adults. Sleep Med. Clin. 13, 93–106. doi: 10.1016/j.jsmc.2017.09.009, PMID: 29412987 PMC5841581

[ref18] Eyileten C. Sharif L. Wicik Z. Jakubik D. Jarosz-Popek J. Soplinska A.. (2021). The relation of the brain-derived neurotrophic factor with microRNAs in neurodegenerative diseases and ischemic stroke. Mol. Neurobiol. 58, 329–347. doi: 10.1007/s12035-020-02101-2, PMID: 32944919 PMC7695657

[ref19] Gao H. M. Hong J. S. (2008). Why neurodegenerative diseases are progressive: uncontrolled inflammation drives disease progression. Trends Immunol. 29, 357–365. doi: 10.1016/j.it.2008.05.002, PMID: 18599350 PMC4794280

[ref20] Gelb A. W. Bayona N. A. Wilson J. X. Cechetto D. F. (2002). Propofol anesthesia compared to awake reduces infarct size in rats. Anesthesiology 96, 1183–1190. doi: 10.1097/00000542-200205000-00023, PMID: 11981160

[ref21] Germann A. L. Shin D. J. Manion B. D. Edge C. J. Smith E. H. Franks N. P.. (2016). Activation and modulation of recombinant glycine and GABA_A_ receptors by 4-halogenated analogues of propofol. Br. J. Pharmacol. 173, 3110–3120. doi: 10.1111/bph.13566, PMID: 27459129 PMC5056230

[ref22] Han R. Huang H. Han H. Chen H. Zeng F. Xie X.. (2021). Propofol postconditioning ameliorates hypoxia/reoxygenation induced H9c2 cell apoptosis and autophagy via upregulating forkhead transcription factors under hyperglycemia. Mil. Med. Res. 8, 1–16. doi: 10.1186/s40779-021-00353-0 34753510 PMC8579603

[ref23] Hara M. Kai Y. Ikemoto Y. (1994). Enhancement by propofol of the gamma-aminobutyric acidA response in dissociated hippocampal pyramidal neurons of the rat. Anesthesiology 81, 988–994. doi: 10.1097/00000542-199410000-00026, PMID: 7943850

[ref24] Hoyer-Hansen M. Bastholm L. Szyniarowski P. Campanella M. Szabadkai G. Farkas T.. (2007). Control of macroautophagy by calcium, calmodulin-dependent kinase kinase-beta, and Bcl-2. Mol. Cell 25, 193–205. doi: 10.1016/j.molcel.2006.12.009, PMID: 17244528

[ref25] Hsing C. H. Chou W. Wang J. J. Chen H. W. Yeh C. H. (2011). Propofol increases bone morphogenetic protein-7 and decreases oxidative stress in sepsis-induced acute kidney injury. Nephrol. Dial. Transplant. 26, 1162–1172. doi: 10.1093/ndt/gfq572, PMID: 20864551

[ref26] Hylin M. J. Zhao J. Tangavelou K. Rozas N. S. Hood K. N. MacGowan J. S.. (2018). A role for autophagy in long-term spatial memory formation in male rodents. J. Neurosci. Res. 96, 416–426. doi: 10.1002/jnr.24121, PMID: 29230855 PMC6425965

[ref27] Jayaraj R. L. Azimullah S. Beiram R. Jalal F. Y. Rosenberg G. A. (2019). Neuroinflammation: friend and foe for ischemic stroke. J. Neuroinflammation 16:142. doi: 10.1186/s12974-019-1516-2 31291966 PMC6617684

[ref28] Jin R. Liu H. Jin W. Shi J. Jin Q. Chu C.. (2015). Propofol depresses cerebellar Purkinje cell activity via activation of GABA_A_ and glycine receptors *in vivo* in mice. Eur. J. Pharmacol. 764, 87–93. doi: 10.1016/j.ejphar.2015.06.052, PMID: 26142083

[ref29] Jin Y. C. Kim W. Ha Y. M. Shin I. W. Sohn J. T. Kim H. J.. (2009). Propofol limits rat myocardial ischemia and reperfusion injury with an associated reduction in apoptotic cell death *in vivo*. Vasc. Pharmacol. 50, 71–77. doi: 10.1016/j.vph.2008.10.002 18996224

[ref30] Kim V. N. (2005). Small RNAs: classification, biogenesis, and function. Mol. Cells 19, 1–15. PMID: 15750334

[ref31] Kloosterman W. P. Plasterk R. H. (2006). The diverse functions of microRNAs in animal development and disease. Dev. Cell 11, 441–450. doi: 10.1016/j.devcel.2006.09.009 17011485

[ref32] Koch S. Rupp L. Prager C. Mörgeli R. Kramer S. Wernecke K. D.. (2018). Incidence of epileptiform discharges in children during induction of anaesthesia using propofol versus sevoflurane. Clin. Neurophysiol. 129, 1642–1648. doi: 10.1016/j.clinph.2018.05.013, PMID: 29913339

[ref33] Landgraf P. Rusu M. Sheridan R. Sewer A. Iovino N. Aravin A.. (2007). A mammalian microRNA expression atlas based on small RNA library sequencing. Cells 129, 1401–1414. doi: 10.1016/j.cell.2007.04.040, PMID: 17604727 PMC2681231

[ref34] Li F. Wang Y. Zheng K. (2023). Microglial mitophagy integrates the microbiota-gut-brain axis to restrain neuroinflammation during neurotropic herpesvirus infection. Autophagy 19, 734–736. doi: 10.1080/15548627.2022.2102309, PMID: 35849507 PMC9851194

[ref35] Li L. Zhang Q. Tan J. Fang Y. An X. Chen B. (2014). Autophagy and hippocampal neuronal injury. Sleep Breath. 18, 243–249. doi: 10.1007/s11325-013-0930-4 24402351

[ref36] Li P. Hao X. C. Luo J. Lv F. Wei K. Min S. (2016). Propofol mitigates learning and memory impairment after electroconvulsive shock in depressed rats by inhibiting autophagy in the hippocampus. Med. Sci. Monit. 22, 1702–1708. doi: 10.12659/msm.897765, PMID: 27203836 PMC4917309

[ref37] Li X. Li W. Luo J. Wei K. Li P. Liu X. B.. (2012). Effects of propofol on the activation of hippocampal CaMKIIα in depressed rats receiving electroconvulsive therapy. J. ECT 28, 242–247. doi: 10.1097/YCT.0b013e31826140c7, PMID: 23041766

[ref38] Li Y. He Z. Lv H. Chen W. Chen J. (2020). Calpain-2 plays a pivotal role in the inhibitory effects of propofol against TNF-α-induced autophagy in mouse hippocampal neurons. J. Cell. Mol. Med. 24, 9287–9299. doi: 10.1111/jcmm.15577, PMID: 32627970 PMC7417688

[ref39] Liang C. Cang J. Wang H. Xue Z. (2013). Propofol attenuates cerebral ischemia/reperfusion injury partially using heme oxygenase-1. J. Neurosurg. Anesthesiol. 25, 311–316. doi: 10.1097/ANA.0b013e31828c6af5, PMID: 23519372

[ref40] Liang X. H. Jackson S. Seaman M. Brown K. Kempkes B. Hibshoosh H.. (1999). Induction of autophagy and inhibition of tumorigenesis by beclin 1. Nature 402, 672–676. doi: 10.1038/45257 10604474

[ref41] Liu C. Peng S. Li Q. (2019). RE-1 silencing transcription factor alleviates the growth-suppressive effects of propofol on mouse neuronal cells. Neuroreport 30, 1025–1030. doi: 10.1097/WNR.0000000000001321, PMID: 31503207

[ref42] Liu H. Dai C. Fan Y. Guo B. Ren K. Sun T.. (2017). From autophagy to mitophagy: the roles of P62 in neurodegenerative diseases. J. Bioenerg. Biomembr. 49, 413–422. doi: 10.1007/s10863-017-9727-7 28975445

[ref43] Liu H. Qu X. Yin X. Li J. Cao Y. Wang Y.. (2022). Intestinal microbiome and metabolome changes induced by sevoflurane, propofol, and sevoflurane-propofol anaesthesia in patients undergoing nephrectomy. Br. J. Anaesth. 129, e38–e40. doi: 10.1016/j.bja.2022.04.028, PMID: 35725658

[ref44] Liu X. Yang B. Tan Y. Feng J. Jia J. Yang C.. (2022). The role of AMPK-Sirt1-autophagy pathway in the intestinal protection process by propofol against regional ischemia/reperfusion injury in rats. Int. Immunopharmacol. 111:109114. doi: 10.1016/j.intimp.2022.109114, PMID: 35933747

[ref45] Logan S. Jiang C. Yan Y. Inagaki Y. Arzua T. Bai X. (2018). Propofol alters long non-coding RNA profiles in the neonatal mouse hippocampus: implication of novel mechanisms in anesthetic-induced developmental neurotoxicity. Cell. Physiol. Biochem. 49, 2496–2510. doi: 10.1159/000493875, PMID: 30261491 PMC6221186

[ref46] Margolis K. G. Gershon M. D. (2016). Enteric neuronal regulation of intestinal inflammation. Trends in Neurosci. 39, 614–624. doi: 10.1016/j.tins.2016.06.007, PMID: 27450201 PMC5002370

[ref47] Menzies F. M. Fleming A. Caricasole A. Bento C. F. Andrews S. P. Ashkenazi A.. (2017). Autophagy and neurodegeneration: pathogenic mechanisms and therapeutic opportunities. Neuron 93, 1015–1034. doi: 10.1016/j.neuron.2017.01.022, PMID: 28279350

[ref48] Mizushima N. Ohsumi Y. Yoshimori T. (2002). Autophagosome formation in mammalian cells. Cell Struct. Funct. 27, 421–429. doi: 10.1247/csf.27.421 12576635

[ref49] Ning H. Yuan H. Xu H. He X. (2017). Propofol reduces hypoxia-induced autophagic cell death through downregulating HIF 1α in alveolar epithelial type II cells of rats. Mol. Med. Rep. 16, 1509–1515. doi: 10.3892/mmr.2017.6697, PMID: 28586054

[ref50] Oh Y. M. Lee S. W. Yoo A. S. (2023). Modeling Huntington disease through microRNA-mediated neuronal reprogramming identifies age-associated autophagy dysfunction driving the onset of neurodegeneration. Autophagy 19, 2613–2615. doi: 10.1080/15548627.2023.2175572, PMID: 36727408 PMC10392748

[ref51] Ohshita N. Gamoh S. Kanazumi M. Nakajima M. Momota Y. Tsutsumi Y. M. (2017). Anesthetic management of a patient with multiple sclerosis. Anesth. Prog. 64, 97–101. doi: 10.2344/anpr-64-02-10, PMID: 28604090 PMC5467764

[ref52] Otabe H. Nibuya M. Shimazaki K. Toda H. Suzuki G. Nomura S.. (2014). Electroconvulsive seizures enhance autophagy signaling in rat hippocampus. Prog. Neuro-Psychopharmacol. Biol. Psychiatry 50, 37–43. doi: 10.1016/j.pnpbp.2013.11.012, PMID: 24316174

[ref53] Pagnin D. de Queiroz V. Pini S. Cassano G. B. (2004). Efficacy of ECT in depression: a meta-analytic review. J. ECT 20, 13–20. doi: 10.1097/00124509-200403000-00004 15087991

[ref54] Park H. Kang J. H. Lee S. (2020). Autophagy in neurodegenerative diseases: a hunter for aggregates. Int. J. Mol. Sci. 21:3369. doi: 10.3390/ijms21093369, PMID: 32397599 PMC7247013

[ref55] Pattingre S. Levine B. (2006). Bcl-2 inhibition of autophagy: a new route to cancer? Cancer Res. 66, 2885–2888. doi: 10.1158/0008-5472.CAN-05-4412, PMID: 16540632

[ref56] Pigot M. Andrade C. Loo C. (2008). Pharmacological attenuation of electroconvulsive therapy-induced cognitive deficits: theoretical background and clinical findings. J. ECT 24, 57–67. doi: 10.1097/YCT.0b013e3181616c14 18379337

[ref57] Qiao H. Li Y. Xu Z. Li W. Fu Z. Wang Y.. (2017). Propofol affects neurodegeneration and neurogenesis by regulation of autophagy via effects on intracellular calcium homeostasis. Anesthesiology 127, 490–501. doi: 10.1097/ALN.0000000000001730, PMID: 28614084 PMC5561483

[ref58] Rabelo F. A. Kupper D. S. Sander H. H. Fernandes R. M. Valera F. C. (2013). Polysomnographic evaluation of propofol-induced sleep in patients with respiratory sleep disorders and controls. Laryngoscope 123, 2300–2305. doi: 10.1002/lary.23664, PMID: 23801248

[ref59] Salter M. W. Stevens B. (2017). Microglia emerge as central players in brain disease. Nat. Med. 23, 1018–1027. doi: 10.1038/nm.4397 28886007

[ref60] Sheridan D. C. Spiro D. M. Nguyen T. Koch T. K. Meckler G. D. (2012). Low-dose propofol for the abortive treatment of pediatric migraine in the emergency department. Pediatr. Emerg. Care 28, 1293–1296. doi: 10.1097/PEC.0b013e3182768a6b, PMID: 23187986

[ref61] Shi S. S. Zhang H. B. Wang C. H. Yang W. Z. Liang R. S. Chen Y.. (2015). Propofol attenuates early brain injury after subarachnoid hemorrhage in rats. J. Mol. Neurosci. 57, 538–545. doi: 10.1007/s12031-015-0634-2, PMID: 26342279

[ref62] Shiratsuchi H. Kouatli Y. Yu G. X. Marsh H. M. Basson M. D. (2009). Propofol inhibits pressure-stimulated macrophage phagocytosis via the Gabaa receptor and dysregulation of p130cas phosphorylation. Am. J. Physiol. 296, C1400–C1410. doi: 10.1152/ajpcell.00345.2008, PMID: 19357231 PMC2692417

[ref63] Shoair O. A. Grasso I. M. Lahaye L. A. Daniel R. Biddle C. J. Slattum P. W. (2015). Incidence and risk factors for postoperative cognitive dysfunction in older adults undergoing major noncardiac surgery: a prospective study. J. Anaesthesiol. Clin. Pharmacol. 31, 30–36. doi: 10.4103/0970-9185.150530, PMID: 25788770 PMC4353149

[ref64] Singh A. Kukreti R. Saso L. Kukreti S. (2019). Oxidative stress: a key modulator in neurodegenerative diseases. Molecules 24:1583. doi: 10.3390/molecules24081583, PMID: 31013638 PMC6514564

[ref65] Smaili S. S. Pereira G. J. Costa M. M. Rocha K. K. Rodrigues L. Do C. L.. (2013). The role of calcium stores in apoptosis and autophagy. Curr. Mol. Med. 13, 252–265. doi: 10.2174/156652413804810772, PMID: 23228221

[ref66] Soliman R. Mofeed M. Momenah T. (2017). Propofol versus ketofol for sedation of pediatric patients undergoing transcatheter pulmonary valve implantation: a double-blind randomized study. Ann. Card. Anaesth. 20, 313–317. doi: 10.4103/aca.ACA_24_17, PMID: 28701596 PMC5535572

[ref67] Spiller H. A. Hays H. L. Aleguas A. (2013). Overdose of drugs for attention-deficit hyperactivity disorder: clinical presentation, mechanisms of toxicity, and management. CNS Drugs 27, 531–543. doi: 10.1007/s40263-013-0084-8, PMID: 23757186

[ref68] Sun B. Ou H. Ren F. Guan Y. Huan Y. Cai H. (2021). Propofol protects against cerebral ischemia/reperfusion injury by down-regulating long noncoding RNA SNHG14. ACS Chem. Neurosci. 12, 3002–3014. doi: 10.1021/acschemneuro.1c00059, PMID: 34369750

[ref69] Sun B. Ou H. Ren F. Huan Y. Zhong T. Gao M.. (2018). Propofol inhibited autophagy through Ca^2+^/CaMKKβ/AMPK/mTOR pathway in OGD/R-induced neuron injury. Mol. Med. 24:58. doi: 10.1186/s10020-018-0054-1, PMID: 30470173 PMC6251140

[ref70] Sweni S. Meenakshisundaram R. Senthilkumaran S. Thirumalaikolundusubramanian P. (2011). Propofol’s derivative: a potential drug for erectile dysfunction? Med. Hypotheses 77, 668–670. doi: 10.1016/j.mehy.2011.07.011, PMID: 21802862

[ref71] Tanida I. Ueno T. Kominami E. (2008). LC3 and autophagy. Methods Mol. Biol. 445, 77–88. doi: 10.1007/978-1-59745-157-4_4 18425443

[ref72] Uzdensky A. B. (2019). Apoptosis regulation in the penumbra after ischemic stroke: expression of pro- and antiapoptotic proteins. Apoptosis 24, 687–702. doi: 10.1007/s10495-019-01556-6, PMID: 31256300

[ref73] Vansant G. Trauger R. J. Cameron A. Vendemelio M. Kreitschitz S. Carlo A. T.. (2009). Propofol hemisuccinate suppression of experimental autoimmune encephalomyelitis. Autoimmunity 40, 180–186. doi: 10.1080/08916930701204467, PMID: 17453716

[ref74] Vutskits L. Xie Z. (2016). Lasting impact of general anaesthesia on the brain: mechanisms and relevance. Nat. Rev. Neurosci. 17, 705–717. doi: 10.1038/nrn.2016.128, PMID: 27752068

[ref75] Walsh E. C. Lee J. M. Terzakis K. Zhou D. W. Burns S. Buie T. M.. (2018). Age-dependent changes in the propofol-induced electroencephalogram in children with autism spectrum disorder. Front. Syst. Neurosci. 12:23. doi: 10.3389/fnsys.2018.00023, PMID: 29988455 PMC6024139

[ref76] Wang B. Shravah J. Luo H. Raedschelders K. Chen D. D. Ansley D. M. (2009). Propofol protects against hydrogen peroxide-induced injury in cardiac H9c2 cells via Akt activation and Bcl-2 up-regulation. Biochem. Biophys. Res. Commun. 389, 105–111. doi: 10.1016/j.bbrc.2009.08.097, PMID: 19703415 PMC3631547

[ref77] Wang H. Zheng S. Liu M. Jia C. Wang S. Wang X.. (2016). The effect of propofol on mitochondrial fission during oxygen-glucose deprivation and reperfusion injury in rat hippocampal neurons. PLoS One 11:e165052. doi: 10.1371/journal.pone.0165052, PMID: 27788177 PMC5082830

[ref78] Wang Y. Tian D. Wei C. Cui V. Wang H. Zhu Y.. (2020). Propofol attenuates alpha-synuclein aggregation and neuronal damage in a mouse model of ischemic stroke. Neurosci. Bull. 36, 289–298. doi: 10.1007/s12264-019-00426-0, PMID: 31520398 PMC7056784

[ref79] Wehrmann T. Triantafyllou K. (2010). Propofol sedation in gastrointestinal endoscopy: a gastroenterologist’s perspective. Digestion 82, 106–109. doi: 10.1159/000285554, PMID: 20407257

[ref80] Westphalen R. I. Hemmings H. C. (2003). Effects of isoflurane and propofol on glutamate and GABA transporters in isolated cortical nerve terminals. Anesthesiology 98, 364–372. doi: 10.1097/00000542-200302000-00016 12552195

[ref81] Xiao X. Hou Y. Yu W. Qi S. (2021). Propofol ameliorates microglia activation by targeting microRNA-221/222-IRF2 axis. J Immunol Res 2021, 1–12. doi: 10.1155/2021/3101146, PMID: 34423051 PMC8373515

[ref82] Xie Z. Dong Y. Maeda U. Moir R. D. Xia W. Culley D. J.. (2007). The inhalation anesthetic isoflurane induces a vicious cycle of apoptosis and amyloid beta-protein accumulation. J. Neurosci. 27, 1247–1254. doi: 10.1523/JNEUROSCI.5320-06.2007, PMID: 17287498 PMC6673586

[ref83] Xiu M. Luan H. Gu X. Liu C. Xu D. (2022). MicroRNA-17-5p protects against propofol anesthesia-induced neurotoxicity and autophagy impairment via targeting BCL2L11. Comput. Math. Methods Med. 2022:6018037. doi: 10.1155/2022/6018037, PMID: 35799645 PMC9256336

[ref84] Xu F. Ma R. Zhang G. Wang S. Yin J. Wang E.. (2018). Estrogen and propofol combination therapy inhibits endoplasmic reticulum stress and remarkably attenuates cerebral ischemia-reperfusion injury and OGD injury in hippocampus. Biomed. Pharmacother. 108, 1596–1606. doi: 10.1016/j.biopha.2018.09.167 30372862

[ref85] Xu Q. Guohui M. Li D. Bai F. Fang J. Zhang G.. (2021). lncRNA C2dat2 facilitates autophagy and apoptosis via the miR-30d-5p/DDIT4/mTOR axis in cerebral ischemia-reperfusion injury. Aging 13, 11315–11335. doi: 10.18632/aging.202824, PMID: 33833132 PMC8109078

[ref86] Xu Z. D. Wang Y. Liang G. Liu Z. Q. Ma W. H. Chu C. T.. (2020). Propofol affects mouse embryonic fibroblast survival and proliferation *in vitro* via ATG5-and calcium-dependent regulation of autophagy. Acta Pharmacol. Sin. 41, 303–310. doi: 10.1038/s41401-019-0303-z, PMID: 31645660 PMC7471456

[ref87] Xu Z. Jiang X. Li W. Gao D. Li X. Liu J. (2011). Propofol-induced sleep: efficacy and safety in patients with refractory chronic primary insomnia. Cell Biochem. Biophys. 60, 161–166. doi: 10.1007/s12013-010-9135-7, PMID: 21107748

[ref88] Yang M. Wang Y. Liang G. Xu Z. Chu C. T. Wei H. (2019). Alzheimer’s disease presenilin-1 mutation sensitizes neurons to impaired autophagy flux and propofol neurotoxicity: role of calcium dysregulation. J. Alzheimers Dis. 67, 137–147. doi: 10.3233/JAD-180858, PMID: 30636740 PMC6367936

[ref89] Yang N. Li L. Li Z. Ni C. Cao Y. Liu T.. (2017). Protective effect of dapsone on cognitive impairment induced by propofol involves hippocampal autophagy. Neurosci. Lett. 649, 85–92. doi: 10.1016/j.neulet.2017.04.019, PMID: 28411068

[ref90] Yang N. Li Z. Han D. Mi X. Tian M. Liu T.. (2020). Autophagy prevents hippocampal alpha-synuclein oligomerization and early cognitive dysfunction after anesthesia/surgery in aged rats. Aging 12, 7262–7281. doi: 10.18632/aging.103074, PMID: 32335546 PMC7202547

[ref91] Yu G. Dymond M. Yuan L. Chaturvedi L. S. Shiratsuchi H. Durairaj S.. (2011). Propofol’s effects on phagocytosis, proliferation, nitrate production, and cytokine secretion in pressure-stimulated microglial cells. Surgery 150, 887–896. doi: 10.1016/j.surg.2011.04.002, PMID: 21676422 PMC3837575

[ref92] Yu Y. Hou K. Ji T. Wang X. Liu Y. Zheng Y.. (2021). The role of exosomal microRNAs in central nervous system diseases. Mol. Cell. Biochem. 476, 2111–2124. doi: 10.1007/s11010-021-04053-0 33528706

[ref93] Zhang J. Li Y. (2023). Propofol-induced developmental neurotoxicity: from mechanisms to therapeutic strategies. ACS Chem. Neurosci. 14, 1017–1032. doi: 10.1021/acschemneuro.2c00755 36854650

[ref94] Zhang J. Xia Y. Xu Z. Deng X. (2016, 2016). Propofol suppressed hypoxia/reoxygenation-induced apoptosis in HBVSMC by regulation of the expression of Bcl-2, Bax, Caspase3, Kir6.1, and p-JNK. Oxidative Med. Cell. Longev.:1518738. doi: 10.1155/2016/1518738, PMID: 27057270 PMC4736333

[ref95] Zhang L. Song H. Ding J. Wang D. J. Zhu S. P. Liu C.. (2022). The mechanism of TNF-alpha-mediated accumulation of phosphorylated tau protein and its modulation by propofol in primary mouse hippocampal neurons: role of mitophagy, NLRP3, and p62/Keap1/Nrf2 pathway. Oxidative Med. Cell. Longev. 2022:8661200. doi: 10.1155/2022/8661200, PMID: 35993019 PMC9391138

[ref96] Zhang T. Ji D. Sun J. Song J. Nie L. Sun N. (2021). NPAS4 suppresses propofol-induced neurotoxicity by inhibiting autophagy in hippocampal neuronal cells. Arch. Biochem. Biophys. 711:109018. doi: 10.1016/j.abb.2021.109018 34418347

[ref97] Zhang T. Tian Y. Zheng X. Li R. Hu L. Shui X.. (2023). Activation of transient receptor potential vanilloid 1 ameliorates tau accumulation-induced synaptic damage and cognitive dysfunction via autophagy enhancement. CNS Neurosci. Ther. doi: 10.1111/cns.14432 [Online ahead of print]., PMID: 37641913 PMC10916438

[ref98] Zhong X. Ouyang C. Liang W. Dai C. Zhang W. (2021). (2R,6R)-hydroxynorketamine alleviates electroconvulsive shock-induced learning impairment by inhibiting autophagy. Neuropsychiatr. Dis. Treat. 17, 297–304. doi: 10.2147/NDT.S278422, PMID: 33568909 PMC7868300

[ref99] Zhou H. Y. Jiang F. Cao Z. Shen Q. Y. Feng Y. J. Hou Z. H. (2021). Propofol protects pc12 cells from cobalt chloride-induced injury by mediating mIR-134. Histol. Histopathol. 36, 425–435. doi: 10.14670/HH-18-298, PMID: 33410125

